# Inhibition of Ferroptosis Ameliorates Photoreceptor Degeneration in Experimental Diabetic Mice

**DOI:** 10.3390/ijms242316946

**Published:** 2023-11-29

**Authors:** Sha Gao, Shuang Gao, Yanuo Wang, Na Li, Zijian Yang, Huiping Yao, Yanwei Chen, Yu Cheng, Yisheng Zhong, Xi Shen

**Affiliations:** Department of Ophthalmology, Ruijin Hospital, Shanghai Jiao Tong University School of Medicine, No. 197, Ruijin 2nd Road, Shanghai 200025, China; gaosha_1343@126.com (S.G.);

**Keywords:** ferroptosis, diabetic retinopathy, photoreceptor, ferrostatin-1, lipid peroxidation

## Abstract

Diabetic retinopathy (DR) is a leading cause of vision impairment in the working-age population worldwide. Various modes of photoreceptor cell death contribute to the development of DR, including apoptosis and autophagy. However, whether ferroptosis is involved in the pathogenesis of photoreceptor degeneration in DR is still unclear. High-glucose (HG)-stimulated 661W cells and diabetic mice models were used for in vitro and in vivo experiments, respectively. The levels of intracellular iron, glutathione (GSH), reactive oxygen species (ROS), lipid peroxidation (MDA), and ferroptosis-related proteins (GPX4, SLC7A11, ACSL4, FTH1, and NCOA4) were quantified to indicate ferroptosis. The effect of ferroptosis inhibition was also assessed. Our data showed the levels of iron, ROS, and MDA were enhanced and GSH concentration was reduced in HG-induced 661W cells and diabetic retinas. The expression of GPX4 and SLC7A11 was downregulated, while the expression of ACSL4, FTH1, and NCOA4 was upregulated in the 661W cells cultured under HG conditions and in the photoreceptor cells in diabetic mice. Furthermore, the administration of the ferroptosis inhibitor ferrostatin-1 (Fer-1) obviously alleviated ferroptosis-related changes in HG-cultured 661W cells and in retinal photoreceptor cells in diabetic mice. Taken together, our findings suggest that ferroptosis is involved in photoreceptor degeneration in the development of the early stages of DR.

## 1. Introduction

Diabetic retinopathy (DR) is a severe complication of diabetes and is one of the leading causes of acquired blindness worldwide [1]. Photoreceptors are the most metabolically demanding cells in the retina [2], making them a primary attack target for early disease [3]. Current studies have reported that various forms of photoreceptor cell death, including apoptosis and autophagy, are involved in the retinal neurodegeneration and promote the development of DR [4,5]. However, there are still no effective treatments to prevent irreversible photoreceptor injury in DR, suggesting that the critical mechanisms of photoreceptor degeneration remain elusive.

Ferroptosis is regulated cell death caused by iron accumulation and lipid peroxidation that is characterized by morphological changes in the mitochondria and has a complex regulatory network [6]. It occurs as a result of an imbalance between antioxidant capacity and the production of lipid active oxygen in cells, eventually leading to membrane rupture and cell death. In recent years, ferroptosis has been found to play an important role in the progression of diabetes mellitus and its multiple complications, such as diabetic nephropathy, diabetic neuropathy, diabetic cardiomyopathy, and DR [7]. Previous studies have demonstrated that iron overload [8], glutathione (GSH) depletion [9], and lipid peroxidation product malondialdehyde (MDA) accumulation [10], the critical factors that trigger ferroptosis, promote the development and progression of DR. In addition, high glucose (HG) administration has been confirmed to induce ferroptosis in human retinal capillary endothelial cells [11]. Significant ferroptosis in retinal pigment epithelial (RPE) cells has also been indicated under HG conditions [12] and in early DR [13]. However, whether ferroptosis is involved in diabetes-induced photoreceptor degeneration remains unclear. 

In this study, we observed that ferroptosis was indeed present in photoreceptors after HG treatment and in early DR, resulting from iron accumulation, GSH depletion, increased reactive oxygen species (ROS) and MDA content, mitochondrial shrinkage, downregulation of glutathione peroxidase 4 (GPX4) and solute carrier family 7 member 11 (SLC7A11, also known as xCT) protein expression, and the upregulation of acyl-CoA synthetase long-chain family member 4 (ACSL4), ferritin heavy chain 1 (FTH1), and nuclear receptor coactivator 4 (NCOA4) expression. Furthermore, ferrostatin-1 (Fer-1), a selective inhibitor of ferroptosis, obviously reverted HG-induced ferroptosis-related changes in the photoreceptors, both in vitro and in vivo. Our study suggests that ferroptosis is one of the critical pathways of photoreceptor degeneration under diabetic conditions and should be pursued as a potential therapeutic target for the treatment of DR.

## 2. Results

### 2.1. HG Promotes Ferroptosis in 661W Cells 

**Figure 1:**
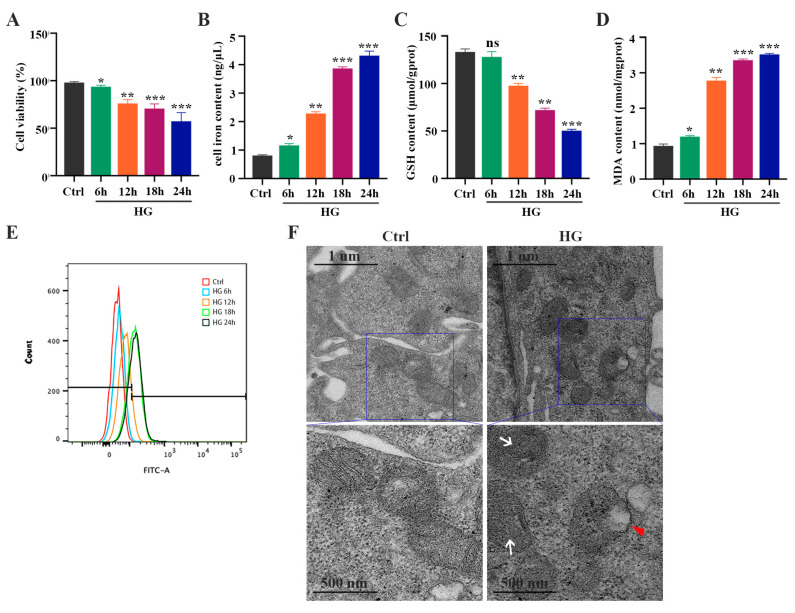
HG induces changes in iron level, GSH concentration, MDA content, and ROS level in 661W cells in a time-dependent manner. (**A**) Cell viability measured via a CCK8 assay revealed a significant increase in the death of 661W cells exposed to 25 mM glucose (HG) in a time-dependent manner. (**B**) Iron level was remarkably increased in HG-stimulated 661W cells compared with the control cells. (**C**) HG induced a marked decrease in GSH concentration after 12, 18, and 24 h. (**D**) HG caused an obvious increase in MDA content in 661W cells after 6, 12, 18, and 24 h. (**E**) Cell ROS level assessed using flow cytometry revealed an increase in HG-stimulated 661W cells after 12, 18, and 24 h. (**F**) Changes in the mitochondrial morphology were detected with TEM in HG-induced 661W cells after 18 h. White arrow indicates mitochondria cristae have vanished. Red triangle indicates outer mitochondria membrane rupture. *p* = not significant [ns], * *p* < 0.05, ** *p* < 0.01, *** *p* < 0.001 versus Ctrl group. Scale bar: 1 μm, 500 nm.

**Figure 2:**
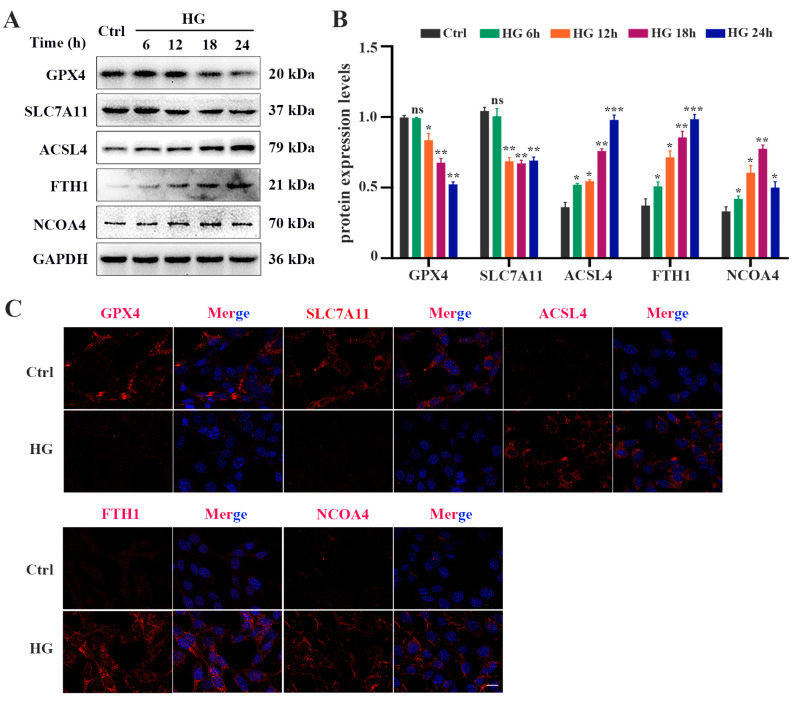
HG causes changes in the expression of ferroptosis-related proteins in 661W cells. (**A**) Western blot analysis of ferroptosis-related protein expression levels in HG-induced 661W cells. GAPDH was used as a control. (**B**) Expression of GPX4 and SLC7A11 proteins was significantly downregulated in HG-stimulated 661W cells after 12, 18, and 24 h. HG induced obvious upregulation in the expression of ACSL4, FTH1, and NCOA4 in 661W cells compared with the Ctrl group. (**C**) Immunofluorescence staining of localization of ferroptosis-related proteins (red) and nuclear (blue) in HG-induced 661W cells after 18 h. Data are shown as mean ± SEM, *n* = 3 per group for Western blotting. *p* = not significant [ns], * *p* < 0.05, ** *p* < 0.01, *** *p* < 0.001 versus Ctrl group. Scale bar: 50 μm.

To evaluate whether HG induces ferroptosis in photoreceptor cells, 661W cells were treated with 25 mM glucose for 6, 12, 18, and 24 h, respectively. The CCK-8 assay results showed that HG treatment could inhibit cell viability in a time-dependent manner (Figure 1A). The amount of total iron (Figure 1B) and lipid peroxidation product MDA (Figure 1D) in the HG-stimulated 661W cells was significantly increased, whereas the GSH level was remarkably decreased in a time-dependent manner (Figure 1C). Meanwhile, we detected elevated levels of ROS in HG-treated 661W cells (Figure 1E). When the mitochondrial morphology was tested, TEM images revealed features of smaller mitochondria with increased membrane density in HG-treated 661W cells at 18 h compared with the control group (Figure 1F). Furthermore, we evaluated the expression levels of various ferroptosis proteins, GPX4, SLC7A11, ACSL4, FTH1, and NCOA4, which are related to lipid peroxidation or iron metabolism. The Western blot analysis results indicated that HG stimulation downregulated the expression of the GPX4 and SLC7A11 proteins but upregulated the ACSL4, FTH1, and NCOA4 protein levels in a time-dependent manner (Figure 2A,B). The immunofluorescence analysis also showed that HG administration remarkably decreased the GPX4 and SLC7A11 expression but increased the amount of ACSL4, FTH1, and NCOA4 in 661W cells at 18 h (Figure 2C).

### 2.2. Fer-1 Alleviates HG-Induced 661W Cell Injury via Inhibiting Ferroptosis

**Figure 3:**
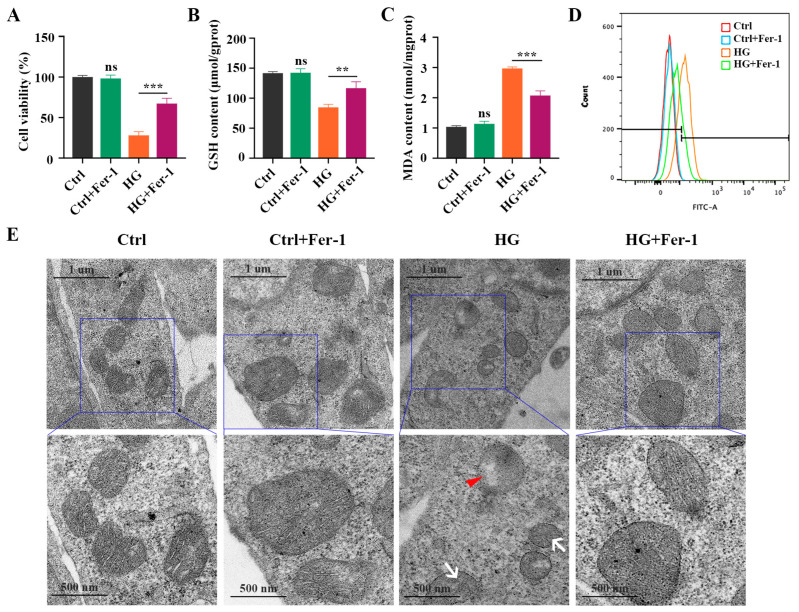
Effect of Fer-1 treatment on GSH concentration, MDA content, and ROS level in HG-stimulated 661W cells after 18 h. (**A**) CCK8 assay showed that Fer-1 treatment significantly inhibited the decrease in cell viability in HG-induced 661W cells. (**B**) The decrease in GSH content in HG-stimulated 661W cells was strongly attenuated after Fer-1 pretreatment. (**C**) The increase in MDA content in HG-stimulated 661W cells was remarkably reduced after Fer-1 administration. (**D**) Fer-1 treatment significantly reversed elevated ROS level in HG-induced 661W cells. (**E**) TEM images of mitochondria structure changes in HG-stimulated 661W cells after Fer-1 treatment. White arrow indicates mitochondria cristae have vanished. Red triangle indicates outer mitochondria membrane rupture. *p* = not significant [ns], ** *p* < 0.01, *** *p* < 0.001 versus Ctrl group. Scale bar: 1 μm, 500 nm.

**Figure 4:**
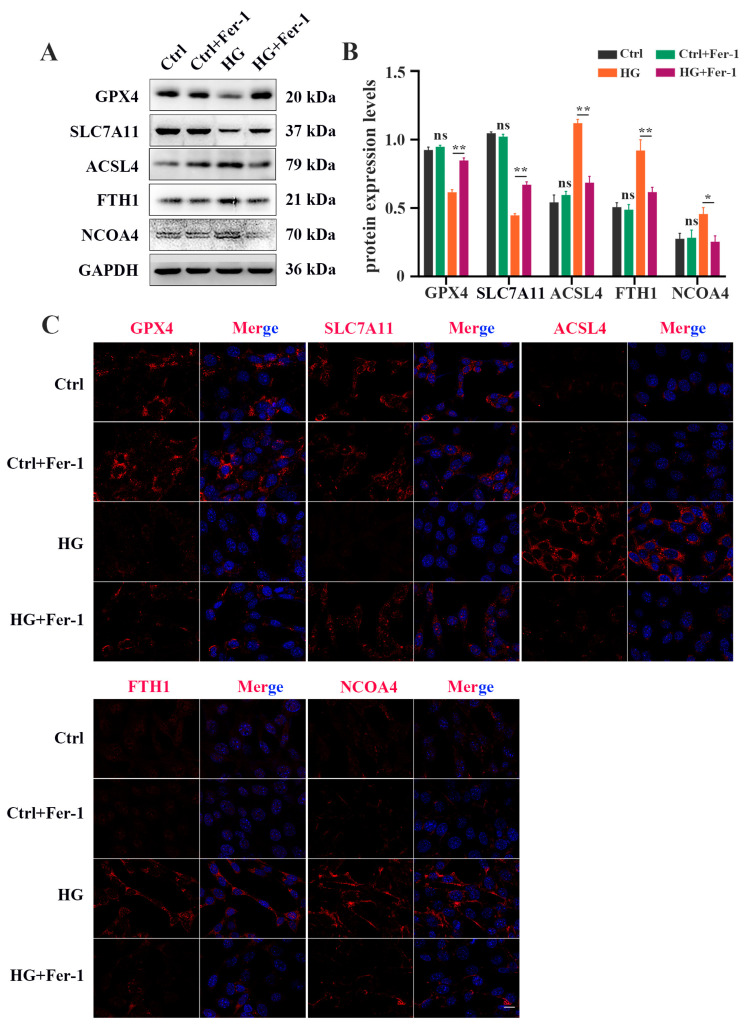
Fer-1 treatment attenuated changes in ferroptosis-related proteins’ expression in HG-stimulated 661W cells after 18 h. (**A**) Western blot analysis of ferroptosis-related proteins’ expression levels in HG-induced 661W cells after Fer-1 treatment. GAPDH was used as a control. (**B**) The downregulation in GPX4 and SLC7A11 protein expression in HG-stimulated 661W cells was significantly attenuated after Fer-1 treatment. The upregulation in ACSL4, FTH1, and NCOA4 protein expression in HG-stimulated 661W cells was effectively abrogated after Fer-1 treatment. (**C**) Immunofluorescence staining of ferroptosis-related proteins (red) and nuclear (blue) in HG-induced 661W cells after Fer-1 administration. Data are shown as mean ± SEM, *n* = 3 per group for Western blotting. *p* = not significant [ns], * *p* < 0.05, ** *p* < 0.01 versus Ctrl group. Scale bar: 50 μm.

Next, we investigated the effect of Fer-1, a selective ferroptosis inhibitor, on HG-induced photoreceptor ferroptosis. Fer-1 pretreatment strongly ameliorated the decrease in the viability of HG-stimulated 661W cells (Figure 3A). The reduced GSH level in the HG-stimulated 661W cells was remarkably increased after Fer-1 treatment (Figure 3B). HG-induced upregulation of MDA content was remarkably inhibited by Fer-1 in the 661W cells (Figure 3C). Meanwhile, Fer-1 administration obviously reversed the elevated ROS level in 661W cells under HG conditions (Figure 3D). Also, these noted mitochondria changes caused by HG were effectively improved by Fer-1 treatment (Figure 3E). In addition, the Western blotting results showed that the decreases in GPX4 and SLC7A11 expression and the increases in ACSL4, FTH1, and NCOA4 protein levels induced by HG were all prevented by Fer-1 administration (Figure 4A,B). Consistent with the Western blotting results, the immunofluorescence analysis further revealed that Fer-1 remarkably alleviated the HG-induced changes in ferroptosis-related proteins’ expression in 661W cells, as evidenced by the increased GPX4 and SLC7A11 expression and the decreased ACSL4, FTH1, and NCOA4 protein levels (Figure 4C).

### 2.3. Ferroptosis Is Involved in Photoreceptor Degeneration in Experimental Diabetic Mice

**Figure 5:**
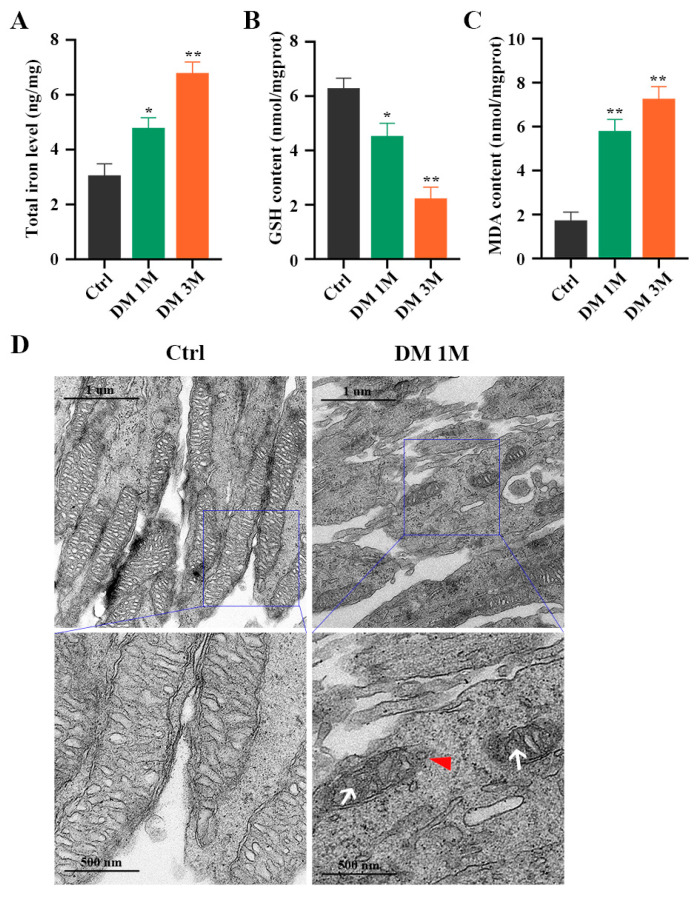
Iron level, GSH concentration, and MDA content in diabetic mice retinas. (**A**) Iron level was markedly increased in retinas at 1 and 3 months post-diabetes. (**B**) A notable decrease was detected in GSH concentration in retinas at 1 and 3 months post-diabetes. (**C**) MDA content was remarkably increased in retinas at 1 and 3 months post-diabetes. (**D**) TEM images showed mitochondria structure changes in retinal photoreceptor cells at 1 month post-diabetes. White arrow indicates mitochondria cristae have vanished. Red triangle indicates outer mitochondria membrane rupture. * *p* < 0.05, ** *p* < 0.01 versus Ctrl group. Scale bar: 1 μm, 500 nm.

**Figure 6:**
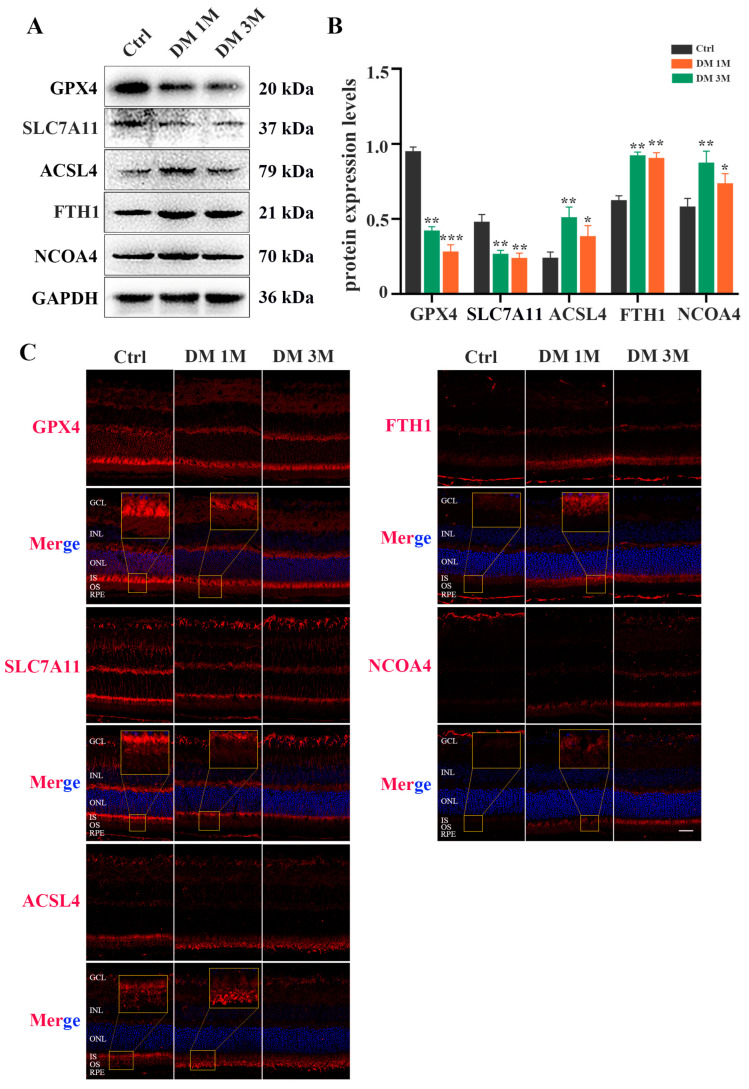
Expression and location of ferroptosis-related proteins in diabetic mice retinas. (**A**) Western blot analysis of ferroptosis-related proteins expression levels in retinas at 1 and 3 months post-diabetes. GAPDH was used as a control. (**B**) GPX4 and SLC7A11 protein expression was remarkably decreased in diabetic mice retinas compared with the control retinas. Expression of ACSL4, FTH1, and NCOA4 proteins was significantly increased in retinas at 1 and 3 months post-diabetes. (**C**) Immunofluorescence staining of location of ferroptosis-related proteins (red) and nuclear (blue) in retinas at 1 and 3 months post-diabetes: ganglion cell layer (GCL), inner nuclear layer (INL), outer nuclear layer (ONL), inner segment (IS), outer segment (OS). Data are shown as mean ± SEM, *n* = 3 per group for Western blotting. * *p* < 0.05, ** *p* < 0.01, *** *p* < 0.001 versus Ctrl group. Scale bar: 50 μm.

To confirm whether ferroptosis contributes to diabetes-induced photoreceptor degeneration, we collected mouse retinas at 1 month and 3 months after diabetes induction. The total iron level (Figure 5A) and MDA content (Figure 5C) were strongly increased, and the GSH concentration was significantly decreased (Figure 5B), in the retinas of STZ-induced diabetic mice compared with the control retinas. Moreover, TME images showed mitochondria changes, with mitochondria cristae vanishing and the outer mitochondria membrane rupturing in the retinas of 1 month post-diabetic mice (Figure 5D). The Western blotting results demonstrated that, compared with the control retinas, low expressions of GPX4 and SLC7A11 proteins and high expressions of ACSL4, FTH1, and NCOA4 proteins were detected in the diabetic retinas (Figure 6A,B). Furthermore, to investigate the location of ferroptosis-related proteins in the diabetic retinas, immunofluorescence staining was performed (Figure 6C). The GPX4 staining was obviously detected in the outer nuclear layer (ONL) and the inner segment (IS) in the control mice, but was remarkably decreased in the diabetic mice. The SLC7A11 immunoreactivity was marked and extended across the entire neural retina to the IS in the control mice, whereas the expression of SLC7A11, especially in the IS, was simultaneously downregulated in the retinas at 1 and 3 months post-diabetes. Compared with the ACSL4 expression that was weakly observed in the IS of the control retinas, the ACSL4 immunoreactivity was remarkably detected in the outer segment (OS) and was accompanied by diminished expression in the IS of the diabetic retinas. The FTH1 and NCOA4 staining had extremely low expression in the control retinas, whereas they both had obviously increased in the IS of the diabetic retinas. These results indicate that ferroptosis is involved in photoreceptor degeneration as early as 1 month after diabetes induction.

### 2.4. Fer-1 Protects Photoreceptors from Ferroptosis in Experimental Diabetic Mice

**Figure 7:**
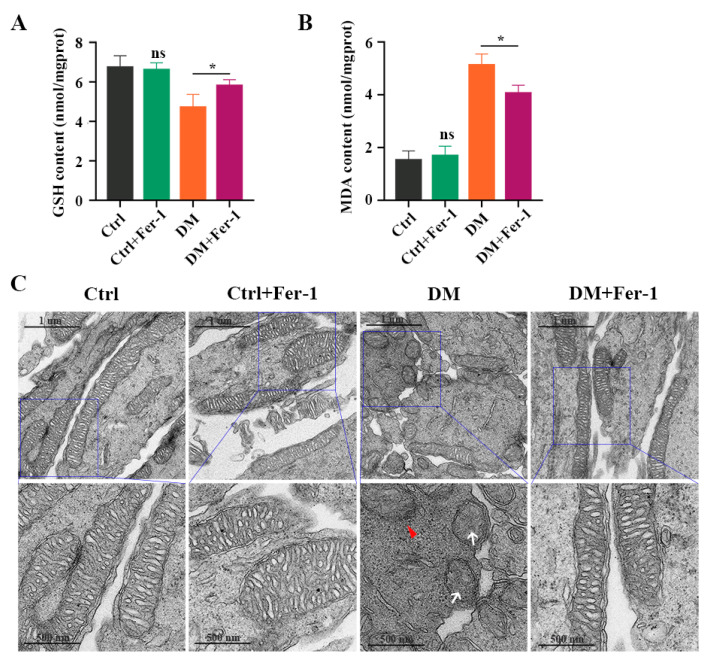
The effect of Fer-1 administration on GSH and MDA content in diabetic mice retinas. (**A**) The decrease in GSH content and (**B**) The increase in MDA content in retinas at 1 month post-diabetes were remarkably attenuated after Fer-1 treatment. (**C**) TEM images of mitochondria structure changes in retinas at 1 month post-diabetes after Fer-1 treatment. White arrow indicates mitochondria cristae have vanished. Red triangle indicates outer mitochondria membrane rupture. *p* = not significant [ns], * *p* < 0.05 versus Ctrl group. Scale bar: 1 μm, 500 nm.

**Figure 8:**
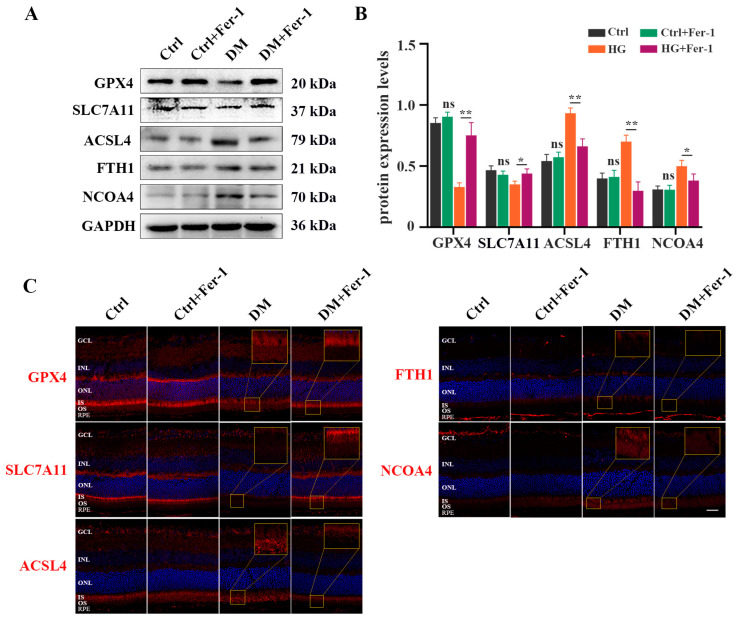
Fer-1 administration attenuated changes of ferroptosis-related proteins expression in diabetic mice retinas. (**A**) Western blot analysis of ferroptosis-related proteins expression levels in diabetic mice retinas after Fer-1 treatment. GAPDH was used as a control. (**B**) The decrease in GPX4 and SLC7A11 protein expression in diabetic mice retinas was significantly attenuated after Fer-1 treatment. The increase in ACSL4, FTH1, and NCOA4 protein expression in diabetic mice retinas was effectively abrogated after Fer-1 treatment. (**C**) Immunofluorescence staining of ferroptosis-related proteins (red) and nuclear (blue) in diabetic mice retinas after Fer-1 treatment: ganglion cell layer (GCL), inner nuclear layer (INL), outer nuclear layer (ONL), inner segment (IS), outer segment (OS). Data are shown as mean ± SEM, *n* = 3 per group for Western blotting. *p* = not significant [ns], * *p* < 0.05, ** *p* < 0.01 versus Ctrl group. Scale bar: 50 μm.

To further investigate whether Fer-1 could inhibit photoreceptor ferroptosis in diabetic mice, we detected ferroptosis-related molecules in the retina at 1 month post-diabetes after Fer-1 administration. Consistent with the results of the in vitro experiment, the decrease in GSH concentration (Figure 7A) and the increase in MDA content (Figure 7B) in diabetic mice retinas were effectively ameliorated after Fer-1 treatment. Meanwhile, Fer-1 administration obviously reversed mitochondria changes in the retinal photoreceptors of diabetic mice (Figure 7C). In addition, the Western blotting results showed that the expression of GPX4 and SLC7A11 was increased, whereas the ACSL4, FTH1, and NCOA4 protein levels were significantly decreased in the DM + Fer-1 group compared with the DM group (Figure 8A,B). Meanwhile, the immunofluorescence staining showed that Fer-1 administration effectively upregulated the SLC7A11 and GPX4 protein levels and strongly decreased the ACSL4, FTH1, and NCOA4 intensity in the photoreceptors of the diabetic mice (Figure 8C). Collectively, these results indicated that Fer-1 could alleviate diabetes-induced photoreceptor degeneration via inhibiting ferroptosis, which plays a critical role in the development of DR.

## 3. Discussion

According to our knowledge, this is the first systematic study to investigate the role of ferroptosis in diabetes-induced retinal photoreceptor degeneration. In the present study, we demonstrated that ferroptosis was significantly induced in HG-cultured 661W cells and in the retinal photoreceptors of diabetic mice with iron accumulation, GSH depletion, mitochondrial shrinkage, enhanced MDA and ROS levels, and changes in the expression of ferroptosis-related proteins, and that Fer-1 treatment dramatically abrogated the systemic changes of ferroptosis in HG-stimulated cultured 661W cells and in the retinal photoreceptors of diabetic mice, providing a novel insight into the mechanisms of diabetes-induced photoreceptor degeneration.

Previous studies have indicated that photoreceptor degeneration is a critical event in the development and progression of DR. Multiple modes of cell death, including apoptosis [14] and autophagy [15], have been demonstrated to play an important role in diabetes-induced photoreceptor degeneration. Notably, although therapies targeting the apoptotic and autophagic pathways have displayed significant advantages in protecting the photoreceptors in DR [14,15], photoreceptor cell loss is still sustained, probably because of the participation of other death pathways. Ferroptosis, a form of iron-dependent regulated cell death triggered by excessive lipid peroxidation [6], is involved in diverse pathophysiological states and has been indicated to play a critical role in the pathogenesis of various diabetic complications, such as diabetic nephropathy [16], diabetic cardiomyopathy [17], and diabetic osteoporosis [18]. Recently, significant ferroptosis has been indicated both in human retinal capillary endothelial cells [19] and in RPE cells [12] under HG conditions. However, whether ferroptosis contributes to photoreceptor damage in diabetic retinas has not been investigated. Herein, for the first time, we show that ferroptosis exacerbates photoreceptor degeneration to promote the development of DR.

Iron is an essential element for a variety of metabolic and physiological processes in living organisms. In contrast, iron accumulation is also harmful. This is because the abnormal accumulation of iron leads to the production of large amounts of free radicals, which cause damage to DNA, proteins, and other biological macromolecules. Chaudhary et al. [8] have found that iron accumulation increases in the retinas of type 1 and type 2 mouse models of diabetes and in postmortem retinal samples from human diabetic patients. Although iron overload has also been demonstrated to increase oxidative stress levels and exacerbate neuronal cell death in diabetic mice retinas, the mechanism of the underlying damage remains unclear. Interestingly, the discovery of ferroptosis gives a novel insight into the pathogenic mechanism of iron accumulation in diseases.

ROS accumulation is the critical mechanism leading to ferroptosis. ROS induces lipid peroxidation in phospholipid-containing cell membranes, resulting in the production of MDA, which directly causes cytotoxicity and then induces ferroptosis [20]. Previous studies have demonstrated GSH depletion [21], ROS accumulation, and increased MDA production [10] in the retinas of diabetic rats. Our study showed that ROS content was obviously increased in HG-induced 661W cells. Moreover, elevated concentrations of MDA were detected both in HG-cultured 661W cells and in the retinas of diabetic mice. Furthermore, according to TEM analysis, shrunken mitochondria, the main morphological feature of ferroptosis [6], were observed with increased mitochondrial membrane density and reduced or absent mitochondrial cristae in the in vivo and in vitro models. The above results suggest that ferroptosis is closely related to photoreceptor change in the development of DR.

Different from other cell death processes, ferroptosis is biologically characterized by multiple markers. GPX4 is one of the most important antioxidant enzymes in mammals and is the only known enzyme that can convert phospholipids to hydrogen peroxide [22]. SLC7A11, as an important factor in the ferroptotic process, brings cystine into the cells, where it is further reduced to cysteine [23]. Cysteine is then transformed into GSH, which is critical for the function of GPX4, through the addition of glycine and glutamate [24]. Thus, the decrease in SLC7A11 expression causes less GSH synthesis, reduced GPX4 activity, and ultimately leads to an increase in lipid peroxidation, which is associated with the loss of plasma membrane integrity. Meanwhile, ACSL4 has been identified as a critical determinant of ferroptosis sensitivity by participating in the production of lipid peroxides [25]. Furthermore, FTH1 and NCOA4 make major contributions to the regulation of iron metabolism and are closely associated with the development of ferroptosis. FTH1 mediates the transformation of ferrous iron to iron via its oxidase activity and reduces the free iron levels by facilitating the incorporation of iron into ferritin [26]. NCOA4 contributes to ferroptosis via directly identifying FTH1 and then delivering the ferritin complex to the autophagosomes, where it undergoes lysosomal degradation and iron release [27,28]. Up to now, there are only a limited number of studies investigating changes in GPX4, SLC7A11, ACSL4, FTH1, and NCOA4 protein expression in diabetic conditions. A previous study showed that the expression of xCT was significantly reduced in HG-induced retinal cells and in the rat retina at 3 weeks after STZ injection, accompanied by a marked decrease in the number of xCT-positive cells in the ganglion cell layer (GCL) and the inner nuclear layer (INL) [29]. Chaudhary and colleagues [8] found that heavy-chain and light-chain ferritin levels were both upregulated in the retinas of type 1 and type 2 diabetic mice aged 12 weeks. Meanwhile, HG remarkably promoted ACSL4 protein expression and decreased GPX4 level in mesangial cells [30]. A high expression of NCOA4 induced the degradation of FTH1 and then promoted free iron release, which is a crucial element to cause ferroptosis, in diabetes myocardial ischemia reperfusion injury [31]. Consistent with previous studies, our results indicated not only that GPX4 and SLC7A11 protein expression was remarkably decreased, but also that ACSL4, FTH1, and NCOA4 protein levels were dramatically increased in HG-induced 661W cells and in mouse photoreceptor cells, suggesting that photoreceptor ferroptosis began as early as 1 month after diabetes induction.

Fer-1, a ferroptosis inhibitor, inhibits ferroptosis by preventing lipid peroxidation and then alleviating pathological changes in various diseases. In the study, we also observed that Fer-1 pretreatment obviously alleviated the HG-induced 661W injury and the photoreceptor damage in diabetic mice, suggesting that ferroptosis is an important contributor to photoreceptor damage induced by hyperglycemia. Notably, although our results indicate that ferroptosis is involved in the photoreceptor degeneration in diabetic mice, further investigations are still needed to elucidate the precise mechanism of the diabetes-induced ferroptosis of the photoreceptors.

In summary, our study results reveal for the first time that ferroptosis plays a critical role in photoreceptor degeneration in the diabetic retina, providing new insights into the pathological mechanism of photoreceptor cells’ injury in early DR, and that blocking ferroptosis with Fer-1 protects photoreceptors from the damage of iron deposition and lipid peroxidation. These findings suggest that ferroptosis could be a potential therapeutic strategy for photoreceptor degeneration in DR.

## 4. Materials and Methods

### 4.1. Experimental Animals

We followed the methods previously reported [32]. All animal experiments followed the guidelines of the Association for Research in Vision and Ophthalmology Statement for the Use of Animals in Ophthalmic and Vision Research and were approved by the Shanghai Jiao Tong University School of Medicine Animal Care and Use Committee. Male, 6–8 weeks old, C57BL/6J mice were allowed free access to water in a climate-controlled room with a 12 h light/12 h dark cycle.

### 4.2. Diabetes Mellitus (DM) Model Establishment and Fer-1 Treatment

The diabetes mellitus model was established as described previously [33]. Diabetes was induced with five sequential daily intraperitoneal injections of a freshly prepared solution of streptozotocin (STZ, Sigma, St. Louis, MO, USA) in citrate buffer (pH 4.5) at 60 mg/kg of body weight. Nondiabetic control mice were administered citrate buffer alone. A total of 14 days following the initial STZ administration, the animals showing fasting blood glucose amounts above 300 mg/dL in 3 consecutive days were considered successful diabetic models. Administration of ferroptosis inhibitor was performed 1 h before diabetes induction according to the previous report [34]; 2 μL Fer-1 (30 μM, SML0583, Sigma-Aldrich, Taufkirchen, Germany) or an equal volume of vehicle was injected intravitreally in experimental eyes.

### 4.3. Cell Culture

Immortalized mouse cone-like (661W) cells were cultured in Dulbecco’s modified Eagle’s medium with 10% fetal bovine serum (FBS) and 1% penicillin/streptomycin. Cultures were incubated at 37 °C with 5% CO_2_, and medium was replaced every 2 days. When confluency reached 80%, the growth medium was replaced with serum-reduced medium (0.5% FBS) overnight prior to the experiments. The cells were then exposed to 5.5 mM glucose (control glucose, Ctrl) in serum-reduced medium for 48 h before stimulating with 25 mM glucose (high glucose, HG) [35]. In the experiments using the ferroptosis inhibitor, 661W cells were pretreated with 100 μΜ Fer-1 for 4 h before HG induction [36].

### 4.4. Cell Viability Assay

The Cell Counting Kit-8 (CCK-8) assay (C0037, Beyotime Biotechnology, Shanghai, China) was used to evaluate 661W cell viability according to the manufacturer’s protocol. In brief, 661W cells were seeded into 96-well plates with a concentration of 8 × 10^3^ cells/well. The cells were cultured for 24 h, then treated with HG for different times (6, 12, 18, 24 h) or pretreated with Fer-1 for 4 h followed by the addition of 20 μL of CCK-8 solution directly into the medium and incubation at 37 °C for 2 h. The optical density values were measured with a microplate reader at 450 nm wavelength.

### 4.5. Iron Assay

Intracellular and tissue iron levels were assessed using an iron assay kit (MAK025, Sigma-Aldrich, Taufkirchen, Germany) as reported previously [36]. Cultured cells or mouse retinal tissues were harvested and homogenized in iron assay buffer. All samples were centrifuged at 16,000× *g* for 10 min. A total of 10 μL of supernatant from each sample was added to 90 μL of the iron assay buffer. Subsequently, 5 μL of iron reducer was added to each supernatant. The mixture was incubated for 30 min, and total iron levels were determined using a microplate reader at a wavelength of 593 nm.

### 4.6. GSH and MDA Assay

The intracellular GSH level and MDA content were determined with a GSH assay kit (S0052, Beyotime Biotechnology, Shanghai, China) and MDA assay kit (S0131S, Beyotime Biotechnology, Shanghai, China), respectively. Briefly, the cells or retinal tissues were harvested, lysed, and centrifuged at 10,000× *g* for 10 min at 4 °C. The supernatant was collected and the GSH level and MDA content were measured using the assay kits according to the manufacturer’s instructions. The absorbance was measured at 412 nm for GSH or 532 nm for MDA using a spectrophotometer. The protein concentration of the samples was determined using a BCA protein assay kit.

### 4.7. ROS Detection

Intracellular ROS level was determined using the ROS Assay Kit (E004-1-1, Nanjing Jian cheng Bioengineering Institute, Nanjing, China) according to the manufacturer’s instruction. Cells were incubated in medium containing 10 μM DCFH-DA for 1 h and then harvested using trypsin and mixed with PBS plus 1% BSA. ROS level was then examined using flow cytometry.

### 4.8. Transmission Electron Microscopy (TEM)

The fresh eye tissues with two holes created in the corneas of different groups were fixed in 2.5% glutaraldehyde at 4 °C for 24 h. The retinas were dissected from the RPE-choroid and continued to be fixed in 2.5% glutaraldehyde for 2 h. The 661W photoreceptor cells of different groups were fixed with 2.5% glutaraldehyde at 4 °C for at least 2 h. Then, the retinal tissues or 661W cells were washed with PBS and fixed in osmium acid solution for 2 h. The samples were washed with PBS again, dehydrated with an ethanol gradient, and embedded into an epoxy resin. Next, the samples were cut into 80 nm sections and double-stained using 3% citrate-uranyl acetate. The images of mitochondrial structure changes were acquired with TEM (FEI Talos L120C, Thermo Scientific, Waltham, MA, USA).

### 4.9. Western Blot Analysis

Western blot analysis was performed according to the previous description [32]. Retinas from experimental eyes were dissected from the RPE-choroid. The retina tissues and 661W cells were homogenized and lysed in RIPA buffer. Protein concentrations were calculated with the BCA protein assay kit (P0009, Beyotime Biotechnology, Shanghai, China). The samples were resolved on 10% SDS-PAGE gels and transferred onto PVDF membranes (Millipore, Taufkirchen, Germany). The membranes were incubated overnight at 4 °C with primary antibodies against GPX4 (ab125066, Abcam, London, GB), SLC7A11 (26864-1-AP, Proteintech, Chicago, IL, USA), ACSL4 (NBP2-16401, Novus Biologicals, Littleton, CO, USA), FTH1 (10727-1-AP, Proteintech, Chicago, IL, USA), NCOA4 (DF4255, Affinity Biosciences, Cincinnati, OH, USA) and GAPDH (10494-1-AP, Proteintech, Chicago, IL, USA). After being washed, the membranes were incubated with horseradish peroxidase-conjugated secondary antibodies (7074S, Cell Signaling Technology, Danvers, MA, USA). The protein expression level was determined with densitometric analysis and normalized to the level of GAPDH.

### 4.10. Immunofluorescent Staining

Immunofluorescence of the retinal sections and 661W cells was performed as previously reported [37]. The eyecups were cut into 10 μm thick sections. The cryosections and 661W cells were, respectively, incubated with monoclonal rabbit anti-mouse GPX4 antibody (ab125066, Abcam, London, GB), polyclonal rabbit anti-mouse SLC7A11 antibody (26864-1-AP, Proteintech, Chicago, IL, USA), polyclonal rabbit anti-mouse ACSL4 antibody (NBP2-16401, Novus Biologicals, Littleton, CO, USA), polyclonal rabbit anti-mouse FTH1 antibody (10727-1-AP, Proteintech, Chicago, IL, USA), and polyclonal rabbit anti-mouse NCOA4 antibody (DF4255, Affinity Biosciences, Cincinnati, OH, USA) using overnight incubation at 4 °C. The cryosections or 661W cells were then incubated in Alexa Fluor™ Plus 555-conjugated anti-rabbit IgG (A32732, Thermo Fisher Scientific, Waltham, MA, USA). The sections were finally counterstained with DAPI (C1006, Beyotime Biotechnology, Shanghai, China). Images were captured with a confocal microscope (SP5, Leica Microsystems, Inc., Wetzlar, Germany) with a fixed detection gain for each comparative section.

### 4.11. Statistical Analysis

The data are presented as the mean ± standard deviation (mean ± SD). The differences among the groups were analyzed with Student’s *t*-test or one-way ANOVA according to the normal distribution. All statistical analysis was performed using GraphPad Prism 8.1.1 software (GraphPad Software, Inc., San Diego, CA, USA). Values of *p* < 0.05 were considered statistically significant.

## Data Availability

Data is contained within the article.
